# Electronic Topological Transition in Ag_2_Te at High-pressure

**DOI:** 10.1038/srep14681

**Published:** 2015-09-30

**Authors:** Yuhang Zhang, Yan Li, Yanmei Ma, Yuwei Li, Guanghui Li, Xuecheng Shao, Hui Wang, Tian Cui, Xin Wang, Pinwen Zhu

**Affiliations:** 1 State Key Laboratory of Superhard Materials, College of Physics, Jilin University, Changchun 130012, China

## Abstract

Recently, Ag_2_Te was experimentally confirmed to be a 3D topological insulator (TI) at ambient pressure. However, the high-pressure behaviors and properties of Ag_2_Te were rarely reported. Here, a pressure-induced electronic topological transition (ETT) is firstly found in Ag_2_Te at 1.8 GPa. Before ETT, the positive pressure coefficient of bulk band-gap, which is firstly found in TIs family, is found by both first-principle calculations and *in situ* high-pressure resistivity measurements. The electrical resistivity obtained at room temperature shows a maximum at 1.8 GPa, which is nearly 3.3 times to that at ambient pressure. This result indicates that the best bulk insulating character and topological nature in Ag_2_Te can be obtained at this pressure. Furthermore, the high-pressure structural behavior of Ag_2_Te has been investigated by *in situ* high-pressure synchrotron powder X-ray diffraction technique up to 33.0 GPa. The accurate pressure-induced phase transition sequence is firstly determined as *P*2_1_/*c* → *Cmca* → *Pnma*. It is worth noting that the reported isostructural *P*2_1_/*c* phase is not existed, and the reported structure of *Cmca* phase is corrected by CALYPSO methodology. The second high-pressure structure, a long puzzle to previous reports, is determined as *Pnma* phase. A pressure-induced metallization in Ag_2_Te is confirmed by the results of temperature-dependent resistivity measurements.

It is known that silver telluride crystallizes in the monoclinic system with space group *P*2_1_/*c* (*β*-Ag_2_Te) under ambient conditions^1^,^2^. In recent decades, *β*-Ag_2_Te has attracted significant interest as a promising candidate for thermoelectric material, infrared detection, and magnetic field sensor^3^,^4^,^5^. Besides, *β*-Ag_2_Te was reported as a 3D topological insulator (TI), which acts as insulators in its bulk while has metallic Dirac fermions on its surface^6^,^7^,^8^. Considerable exotic quantum phenomena have been found in TIs such as Majorana fermions, magnetoelectric effect, and quantum anomalous Hall effect^9^,^10^,^11^. On the other hand, pressure has been widely considered as an effective tool for turning crystalline structures and electronic band structures. For example, pressure-induced electronic topological transition (ETT) transforms Sb_2_Se_3_, BiTeI, and As_2_Te_3_ from insulators into TIs^12^,^13^,^14^,^15^,^16^. In particular, pressure is critical to the formation of topological superconductors^17^,^18^,^19^,^20^,^21^. Moreover, it is obvious that the pressure-induced phase transition sequences for silver chalcogenides are very different^22^,^23^,^24^. For Ag_2_S^22^, it experiences a phase transition sequence: *P*2_1_/*n* → *P*2_1_2_1_2_1_ → *P*2_1_/*n*. For Ag_2_Se^23^, it undergoes structural changes: *P*2_1_2_1_2_1_ → *Pnma* → *Cmcm*. For Ag_2_Te^24^, Zhao *et al.* reported that an isostructural *P*2_1_/*c* phase comes at 2.4 GPa and the *Cmca* phase emerges at 2.8 GPa, while the structure of the third high-pressure phase in Ag_2_Te is still unknown. Therefore, it is interesting to further investigate the high-pressure structural behaviors for Ag_2_Te. Besides, in order to meet the technological application of 3D TIs, a good bulk insulating character is necessary. However, so far, only Bi_2_Se_3_ exhibits an increase of the bulk resistivity under compression^25^,^26^,^27^. Therefore, it is important to explore the electrical transport property of *β*-Ag_2_Te by applying pressure, in order to find a better candidate for implementing devices with 3D TIs.

Here, we report an enhanced topological nature and determination of high-pressure crystal structures for Ag_2_Te by *in-situ* high-pressure resistivity measurements up to 28.4 GPa and room-temperature synchrotron angle-dispersive X-ray diffraction (ADXRD) measurements up to 33.0 GPa, using a diamond-anvil cell (DAC), in conjunction with first-principles calculations.

## Results

**Figure 1:**
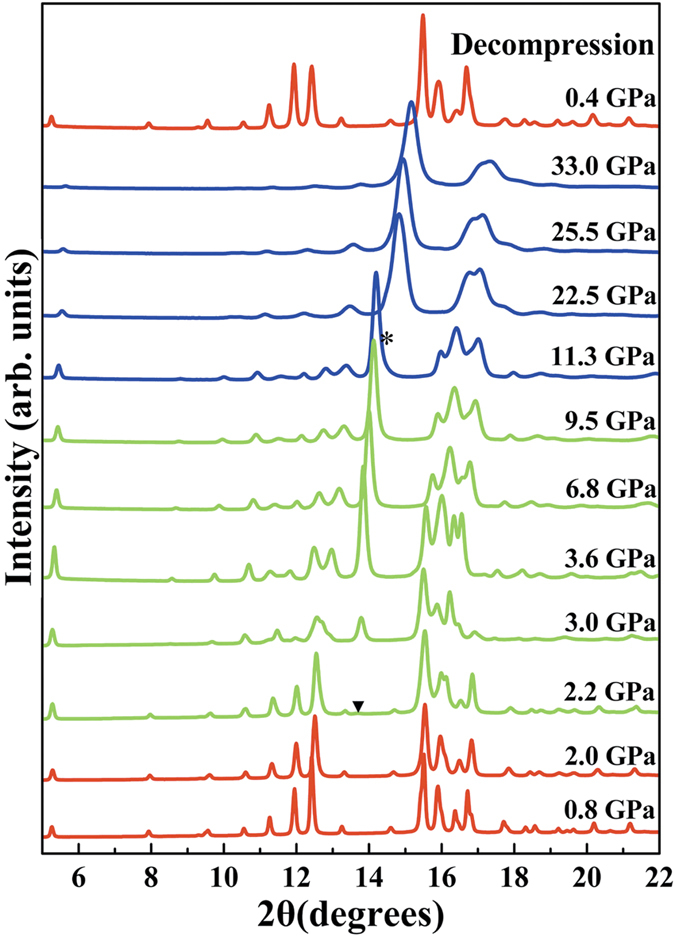
Angle dispersive X-ray powder diffraction patterns of Ag_2_Te under high pressure at room temperature. Arrow and asterisk represent new diffraction peaks.

With increasing pressure, the first and the second pressure-induced structural transitions of Ag_2_Te occur at 2.2 and 11.3 GPa, respectively, which are illustrated in Fig. 1 by the onsets of new peaks. Based on the decompression data, all structural phase transitions are reversible. As can be seen in Supplementary Fig. S1(a,b), Rietveld refinement of ADXRD patterns indicate that the *P*2_1_/*c* phase is retained up to 2.0 GPa. By comparing our Supplementary Fig. S2 with Fig. 1 and Supplementary Fig. S3 in ref. 24, it is clear that our ADXRD patterns of *Cmca* phase are distinct different with those of previous report in intensity sequence of peaks such as (202), (023), (204), and (221) in 3.2–9.5 GPa region. Moreover, a bad fitting result was obtained, when the previously proposed structure of *Cmca* phase was used to carry out Rietveld refinement. So, in order to determine the crystal structure of this phase, the structure prediction via CALYPSO methodology^28^ was performed and a corrected structure of *Cmca* phase was obtained. The corrected structure can result in a good Rietveld fitting (see Supplementary Fig. S3), and the detailed refinement result is shown in Supplementary Table S1. The distinct difference between the corrected structure and the reported structure is mainly in the internal coordinates of atoms. On the other hand, as shown in Supplementary Fig. S4(a), the pattern is well fitted by a combination of *P*2_1_*/c* and *Cmca* phase at 2.2 GPa, and the inset indicates that the (023) characteristic peak of the *Cmca* phase can be observed at 2*θ* = 13.7°, which is ignored by Zhao *et al.* The detailed refinement result for 2.2 GPa are located in Table 1. From Supplementary Fig. S4(b) and the inset of it, it is clear that the first transition is not completed up to 2.6 GPa. Thus, the XRD pattern of 2.4 GPa, measured by previous report, in fact represents mixed structures of *P*2_1_*/c* and *Cmca* phase rather than an isostructural *P*2_1_*/c* phase^24^. When the pressure increase, the second structural transition emerged at 11.3 GPa with a new peak marked at 2*θ* = 14.3°, and the characteristic peak (marked by asterisk) of the second high-pressure phase become gradually stronger as the pressure increases to 19.2 GPa (see Supplementary Fig. S5(a,b)). By the known structures of *A*_2_*B* compounds^23^, the long-puzzling high-pressure phase has been assigned to an orthorhombic structure (space group *Pnma*, No.62). The diffraction data of 25.5 GPa can be well fitted by coexistence of *Cmca* and *Pnma* phase, as shown in Supplementary Fig. S6, and the detailed refinement result can be found in Table 2. The second high-pressure phase transition is not finished up to 33.0 GPa, the highest pressure measured here.

The schematic representation of the high-pressure phase transition sequence for Ag_2_Te is located in Supplementary Fig. S7. It is indicated that the structure of the *P*2_1_/*c* phase is built up of stacking layers of edges-sharing [TeAg8] coordination polyhedron along *a* axis, and the *Cmca* and *Pnma* phase structures can be presented with stacking layers of [TeAg9] coordination polyhedron sharing common faces. The detailed Ag-Te bond lengths of *P*2_1_/*c*, *Cmca* and *Pnma* phase are located in Supplementary Table S2. It is obvious that Ag-Te bond lengths of *Cmca* phase all decreased under pressure. During the transition process from *P*2_1_/*c* phase to *Cmca* phase, Ag1 atoms experience shear glide along *b* axe, leading to the formation of layered rectangle network (see Supplementary Fig. S8(a,b)). As shown in Supplementary Fig. S8(c), the layered Ag1 atom network which are located in *bc* plane undergo shear glide along *c* axe, inducing the layered zigzag network to become flat. It can be seen from Supplementary Fig. S8(d), due to glide takes place in Ag2 atom chain along *b* direction, layered rhombus network of Ag2 atoms are formed when the phase transition occur. As shown in Supplementary Fig. S8(a), thanks to the glide of Ag2 atom chain, the marked Te-Ag2 bond length decreased from 3.896(4) Å to 2.954(6) Å, which result in the [TeAg8] coordination polyhedron developed to [TeAg9] coordination polyhedron via the phase transition. In the second phase transition process, the layered rectangle network of Ag1 atoms become to layered square network, and the layered rhombus network of Ag2 atoms become to layered rectangle network (see Supplementary Fig. S8(a,b,d)). Moreover, the [TeAg9] coordination polyhedron chains undergo shear glide when *Cmca* phase transforms to *Pnma* phase, in Supplementary Fig. S8(c).

**Figure 2:**
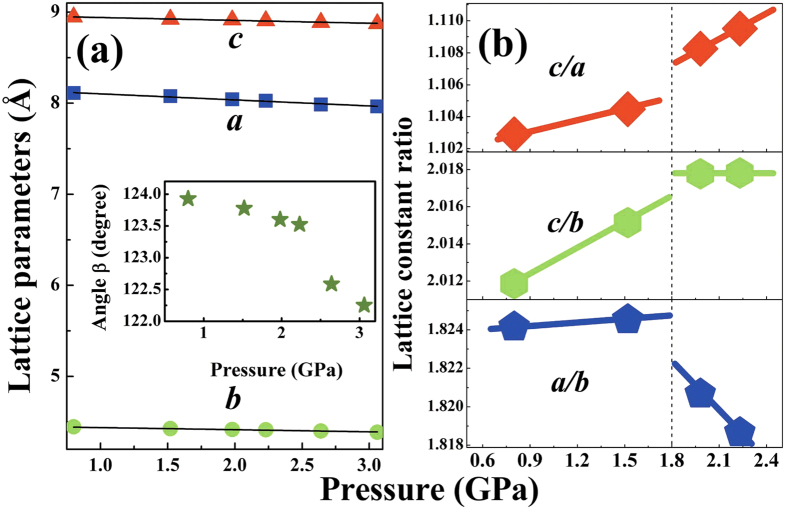
(**a**) Lattice parameters and (**b**) lattice constant ratios as a function of pressure for the *P*2_1_/*c* phase. The solid lines are guide for the eyes. Errors given by the GSAS EXPGUI package are smaller than the marker sizes.

As shown in Fig. 2(a), all the lattice parameters including angle *β* in the *P*2_1_/*c* phase monotonically decrease with increasing pressure. The linear compressibility of the different axes in the *P*2_1_/*c* phase are *κ*_*a*_ = 0.0664(3) GPa^−1^, *κ*_*b*_ = 0.0230(7) GPa^−1^, and *κ*_*c*_ = 0.0314(3) GPa^−1^, respectively. It can be seen that the *b* and *c* axes are less compressible, which is due to Ag1 atoms are all located in *bc* plane, as shown in Supplementary Fig. S8(b), bringing in stronger Ag1-Ag1 interaction. As shown in Fig. 2(b), all the lattice constant ratios display notable changes in compressibility near 1.8 GPa. By taking into account that the ETTs, a modification of the topology of the Fermi surface, are verified by the changes in compressibility of the lattice constant ratios in other TIs—Bi_2_Te_3_, Bi_2_Se_3_ and Sb_2_Te_3_^25^, the above abnormal changes may be ascribed to an ETT around 1.8 GPa.

The presence of active lone electron pairs (LEPs) can result in the asymmetry of coordination polyhedron^12^,^29^. Here, the variance of Ag-Te distances, *K*, is used to quantify the distortion of coordination polyhedron^30^. Based on the Ag-Te distances of *P*2_1_/*c* phase in Supplementary Table S2, the pressure dependence of *K* in *P*2_1_/*c* phase is shown in the Supplementary Fig. S9(a). It can be seen that *K* undergoes an intense fluctuation around the pressure where ETT happens, indicating an increase in the LEP stereochemical activity before 1.8 GPa and the LEP activity experience an intense decrease above that pressure. Therefore, the ETT may be related to the change of the LEP activity from the chalcogen lone-pair *p* orbital^31^ and the existence of the weaker interlayer interaction^32^. As shown in Supplementary Fig. S8(b), Ag1 atoms are located in *ab* plane of *Cmca* phase, which results in stronger Ag1-Ag1 interaction. Therefore, *a* and *b* axes of *Cmca* phase are less compressible than *c* axe, see Supplementary Fig. S9(b), with *κ*_*a*_ = 0.0174(5) GPa^−1^, *κ*_*b*_ = 0.0144(4) GPa^−1^, and *κ*_*c*_ = 0.0336(6) GPa^−1^. This is unlike the previous result that *a* axe was reported more compressible than *b* and *c* axes^24^. As shown in Supplementary Fig. S9(c), the linear compressibility of the different axes in the *Pnma* phase are *κ*_*a*_ = 0.0136(6) GPa^−1^, *κ*_*b*_ = 0.0063(2) GPa^−1^, and *κ*_*c*_ = 0.0118(5) GPa^−1^.

Supplementary Fig. S10 shows the pressure-volume (*P-V*) relationships of the *P*2_1_/*c*, *Cmca* and *Pnma* phase. These *P-V* data are fitted to the usual Birch-Murnaghan (BM equation of state (EOS)^33^.


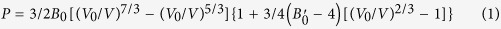


where *B*_*0*_ is the bulk moduli and 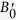 is the pressure derivative. The 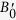 were fixed at 4 for all the phases. We obtain *B*_*0*_ of 66.48(7) GPa (*V*_0_ = 67.78(2) Å^3^) for the *P*2_1_/*c* phase, *B*_*0*_ of 76.89(8) GPa (*V*_0_ = 65.84(0) Å^3^) for the *Cmca* phase, and *B*_*0*_ of 99.03(0) GPa (*V*_0_ = 62.93(9) Å^3^) for the *Pnma* phase. Mulliken population analysis has indicated that the population of Ag-Te covalent bond for *Cmca* phase is larger than that for *P*2_1_/*c* phase, which suggests that the larger *B*_*0*_ of *Cmca* phase comes from the stronger Ag-Te covalent bond. Moreover, it can be seen from Supplementary Table S2, Ag-Te bond length of 2.816(6) Å in *Cmca* phase is smaller than that of *P*2_1_/*c* phase, suggesting that the stronger Ag-Te covalent bond may result from the smaller Ag-Te bond lengths. The bulk moduli of high-pressure phases are different with that of previous report, which is due to the fact that the reported isostructural *P*2_1_/*c* phase is not existed and the reported structure of *Cmca* phase is corrected.

**Figure 3:**
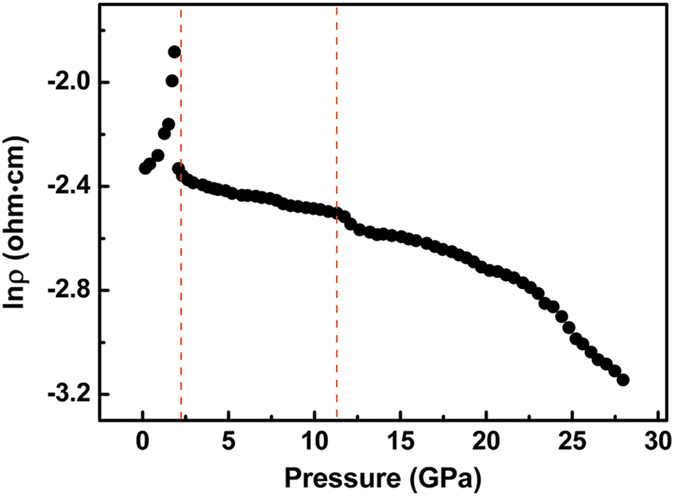
Resistivity as a function of pressure for Ag_2_Te at room temperature.

In order to check on the change of topological nature and the assumption of a pressure-induced ETT in Ag_2_Te near 1.8 GPa, we performs high-pressure resistivity measurements which is believed as an effective supplementary mean for ADXRD measurements to identify electronic structural phase transition. Figure 3 shows the pressure dependence of resistivity for Ag_2_Te at room temperature. The electrical resistivity for Ag_2_Te at 1.8 GPa is nearly 3.3 times to that at ambient pressure, then the electrical resistivity presents a intense collapse and decreases relatively slowly beyond 2.0 GPa. Above ADXRD results have proved that there is no structural transformation until 2.2 GPa. This distinct change may come from ETT^34^. Therefore, in order to shed light on the notable change in the *P*2_1_/*c* phase around 1.8 GPa, we carried out first-principles calculations, which is useful to investigate the effects of pressure-induced ETT on the electronic band structures and may discover the development of topological nature on Ag_2_Te. However, Zhao *et al.* performed first-principles calculation on *P*2_1_/*c* and *Cmca* phase in order to study the pressure-induced metallization.

**Figure 4:**
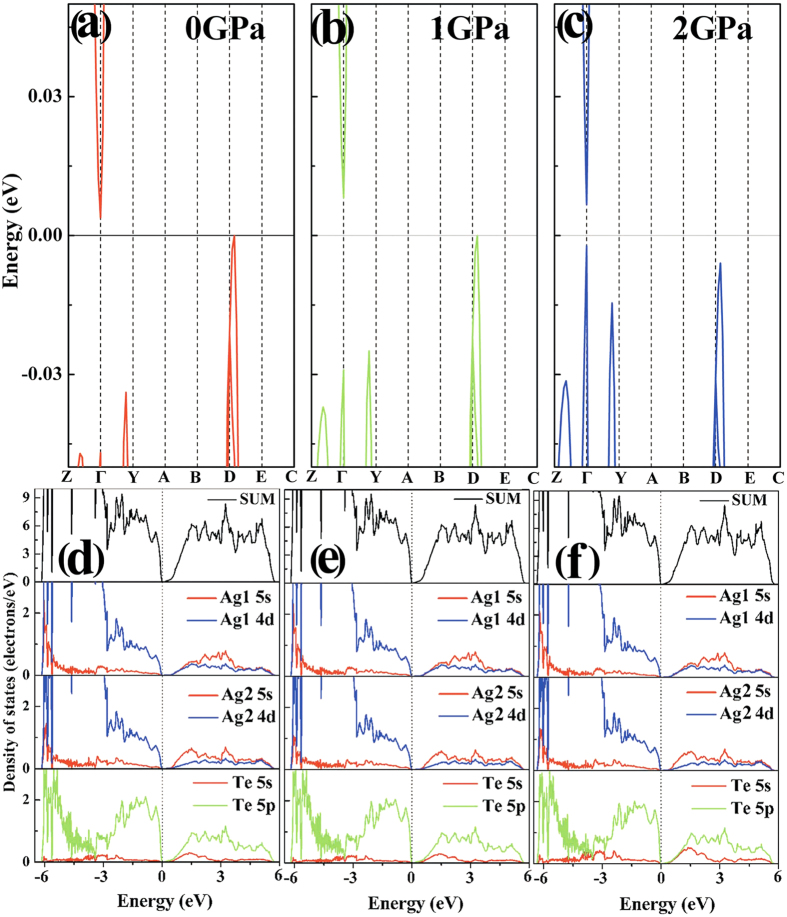
Calculated band structures of Ag_2_Te at (a) ambient pressure, (b) 1.0 GPa, and (c) 2.0 GPa, respectively. Total DOS and PDOS results of Ag_2_Te at (**d**) ambient pressure, (**e**) 1.0 GPa, and (**f**) 2.0 GPa, respectively.

As shown in Fig. 4(a,b), bulk Ag_2_Te is an indirect band-gap semiconductor at ambient pressure and at 1.0 GPa, with the valence-band maximum (VBM) located around *D* point and the conduction-band minimum (CBM) at *Γ* point. However, as shown in Fig. 4c, Ag_2_Te is a direct band-gap semiconductor at 2.0 GPa with VBM and CBM at *Γ* point. At ambient pressure, the band inversion of surface states at *Γ* point is the origin of the topological nature, as discussed by Zhang *et al.*^6^. The partial electron density of state (PDOS) and sum DOS results of Ag_2_Te at ambient pressure, 1.0, and 2.0 GPa, respectively, indicate that VBM are mainly composed by the hybridization of Ag-4*d* and Te-5*p* electrons, as shown in Fig. 4(d–f). The orbital composition is almost invariant under selective pressures. This indicates that the TI character should be stable under pressure^26^. On the other hand, it is found that the increasing interlayer spin-orbit coupling and the fluctuation of LEP activity^35^ under pressure caused a positive pressure coefficient of indirect band-gap and a reduction in the direct band-gap at *Γ* point, which result in an indirect-to-direct transition^36^. Given this indirect-to-direct transformation of Ag_2_Te, the above assumption of a pressure-induced ETT appears reasonable^25^,^37^.

**Figure 5:**
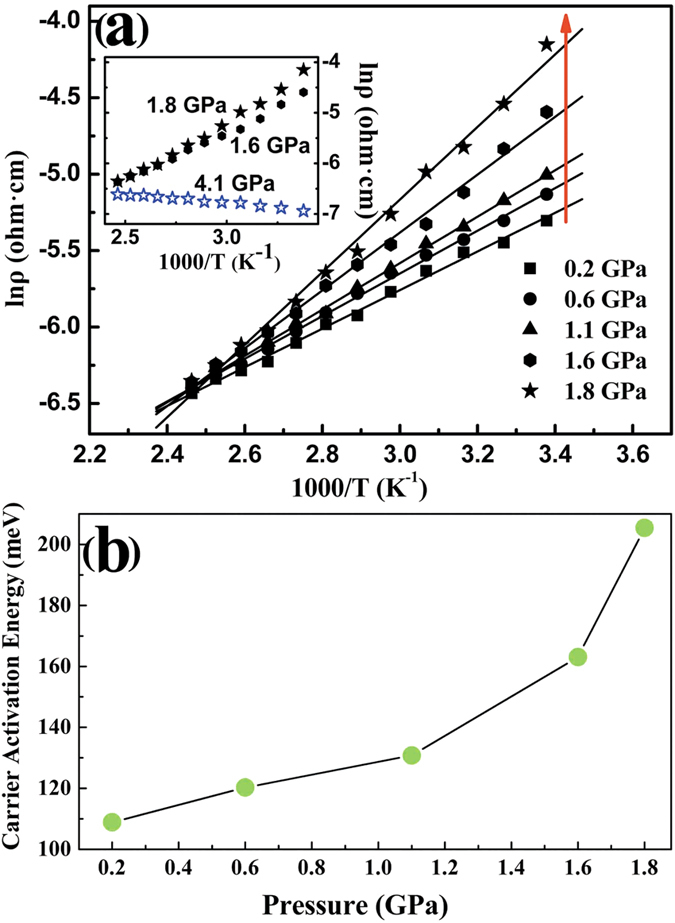
(**a**) Temperature dependence of resistivity for Ag_2_Te. The inset shows resistivity vs temperature at 1.6, 1.8, and 4.1 GPa, respectively. (**b**) Pressure dependence of the carrier activation energy for Ag_2_Te.

Moreover, the resistivity as a function of temperature at various fixed pressures was shown in Fig. 5(a). Before ETT, it is noticed that the resistivity decreases with increasing temperature, demonstrating specific semiconductor behaviors. From Fig. 5(a), the bulk insulating character of Ag_2_Te becomes better and better with increasing pressure, which is a good agreement with our electronic band structure results. As shown in the inset of Fig. 5(a), the resistivity displays a positive relationship with increasing temperature at 4.1 GPa, which implies that the *Cmca* phase performs a metallic behavior. Therefore, the pressure-induced insulator-metal transition was experimentally confirmed by the temperature-dependent resistivity results. The carrier activation energy could be obtained by linearly fitting the plots of ln*ρ* versus 1000/*T*^38^. As shown in Fig. 5(b), there is a continued increase in the carrier activation energy with increasing pressure, indicating a development of carriers energy barriers, which induces that the transport of 3D carrier becomes harder and harder by applying pressure. Therefore, at 1.8 GPa, the best bulk insulating character is obtained, which is the best topological nature of Ag_2_Te by applying pressure.

## Discussion

For applying the technological devices with 3D TIs, one of the most important goals is the control of the 2D electrical conduction in the surface of these materials^25^. Therefore, TIs should exhibit a good bulk insulating character. However, rather high bulk conductivity was observed in most of the TI samples, due to a high concentration of free 3D carriers caused by defects and/or impurities^25^. For Ag_2_Te in this work, before ETT, due to bulk band-gap enhanced under pressure, a decrease of 3D electron concentration can be obtained, resulting in an increase of bulk resistivity, in Fig. 3. Thus, for Ag_2_Te, it is verified that pressure is helpful for suppressing bulk conductivity and revealing the relative contribution of surface states conductivity^25^. So, a better topological nature of Ag_2_Te was obtained by compression. The electronic band structures of Ag_2_Te around ETT are different. And the differences are as follows: (a) The CBM and VBM show higher curvature after ETT, and a lower effective mass is expected^26^. (b) Due to indirect-to-direct band-gap transition, Ag_2_Te becomes a direct band-gap semiconductor after ETT, which is unique for 3D TIs, being a better candidate for infrared detection^4^,^39^. (c) After ETT, the CBM and VBM of bulk states are both located at *Γ* point, which is same as that of surface states, causing a reduction in the separation between surface states and bulk states in the momentum space. However, on the contrary, after ETT, the separation is further enhanced for Bi_2_Te_3_, Bi_2_Se_3_ and Sb_2_Te_3_^20^,^21^,^26^.

In addition, for 3D TIs family, we suggest that the pressures, at which the ETTs occur, are strongly sensitive to carrier concentration and Fermi energy level of the samples, due to charge concentration easily affect the competition between interlayer interaction and LEP activity, which could cause diverse pressure-induced behaviors and properties^25^,^32^,^40^. For example, in previous report, the resistivity of Ag_2−δ_Te decreased continuously as pressure increased to 1.0 GPa, and showed a positive pressure coefficient beyond that pressure^41^. For purchased sample of Sb_2_Se_3_^12^, the Raman study did not reproduce the pressure-induced ETT, but it can be observed in single crystal grown by Bridgeman method^42^. For Bi_2_Se_3_, different authors observed ETT and structural transition at pressures differing by 2–3 GPa^18^,^19^,^26^,^27^,^43^,^44^. In particular, for Bi_2_Te_3_, different hole concentration could give rise to the observations of pressure-induced superconductivity differing by 3–6 GPa^20^,^33^, and the pressure-induced ETT did not appear in n-type samples^45^.

Besides, we would like to mention about the experimental evidences to detect the appearances of pressure-induced ETTs in 3D TIs, as follows. (a) In this work, the pressure-induced ETT is related to the change of the LEP activity which can result in the asymmetry of coordination polyhedron^29^. (b) By means of the changes of the lattice constant ratios, Raman frequencies and Raman linewidths, pressure-induced ETTs have been revealed in Bi_2_Te_3_, Bi_2_Se_3_ and Sb_2_Te_3_, which indicated both XRD measurements and Raman scattering measurements are sensitive methods to evidence the presences of ETTs in this family^25^. (c) Due to the ETTs are shown to strongly influence the thermoelectrical properties of samples, the ETTs can be easily probed by the changes of thermoelectrical properties in 3D TIs^25^,^46^,^47^. Thus, an improvement of thermoelectrical property can be expected in Ag_2_Te after ETT. (d) For both Bi_2_Se_3_ and Ag_2_Te, pressure-induced ETTs can affect the pressure coefficients of resistivity^27^. Based on the change in pressure coefficients of resistivity, we determine that the best topological nature of Ag_2_Te can be seen at 1.8 GPa.

Finally, in order to apply the technological devices with 3D TIs, it is important to obtain a TI with a good topological nature, which has a large band-gap and low bulk charge density^25^. Since, for TIs family, the positive pressure coefficient of bulk band-gap is only found in Ag_2_Te, it is necessary to perform systematic studies on Ag_2_Te under compression, which may provide us an approach of enhancing bulk band-gap of 3D TIs. Thus, some suggestions are as follows: different stoichiometric Ag_2±δ_Te, impurity doping, non-hydrostatic pressures and low temperature measurements could be available experimental plans to induce expected electrical transport properties and TI with a good topological nature under pressure. Angle Resolved Photon Electron Spectroscopy technique should be used to reveal the metallic surface states of Ag_2_Te upon pressure. Low-temperature electrical transport experiments are expected to discover topological superconductor states of Ag_2_Te.

## Methods

### Room-temperature angle-dispersive X-ray diffraction

Ag_2_Te powder was provided by Yuan *et al.*^48^. For high-pressure diffraction measurements, the sample was crushed in a mortar with a pestle. As shown in Supplementary Fig. S11, our diffraction rings pattern is clear. EDX result of powder sample was located in Supplementary Fig. S12. Measurements were performed in a Mao–Bell type DAC with 4/1 methanol/ethanol mixture as the pressure medium. The powder was loaded in a 120 μm diameter hole drilled in the T-301 stainless steel gasket and chips of ruby were added as pressure calibrator^49^. The ADXRD experiments were performed using a synchrotron angle-dispersive x-ray source (*λ* = 0.6199 Å) of the 4W2 High-Pressure Station of Beijing Synchrotron Radiation Facility (BSRF). Patterns were fitted by Rietveld refinement, using the General Structure Analysis System (GSAS) and graphical user interface EXPGUI package^50^.

### High-pressure resistivity measurements

*Van der Pauw* electrodes were integrated on one diamond anvil for *in situ* resistivity measurement under high pressure^51^. The temperature dependence of resistivity measurements was conducted by placing the DAC into a tropical drying cabinet, lasting for more than 10 min to make the thermal balance.

### First-principles calculations

We performed structure prediction through a global minimization of free energy surfaces merging ab initio total-energy calculations via CALYPSO methodology^28^. The geometric optimization were performed using density functional theory with the Perdew–Burke–Ernzerhof exchange–correlation as implemented in the Vienna Ab initio Simulation Package (VASP) code^52^ and the generalized gradient approximation (GGA)^53^ is implemented on a projector augmented wave (PAW) basis^54^,^55^. Integration in the Brillouin zone was performed using special *k* points generated with 5 × 8 × 5 mesh parameter grids. Convergence tests give a kinetic energy cutoff as 550 eV and spin-orbit coupling interaction was included through the calculation.

## Additional Information

**How to cite this article**: Zhang, Y. *et al.* Electronic Topological Transition in Ag_2_Te at High-pressure. *Sci. Rep.* **5**, 14681; doi: 10.1038/srep14681 (2015).

## Supplementary Material

Supplementary Information

## Figures and Tables

**Table 1:** Rietveld refinement results for 2.2 GPa.

Pressure (GPa)	2.2	
Space group	*P*2_1_/*c* (No. 14)	*Cmca*(No. 64)
*a* (Å)	8.0113(1)	6.076(1)
*b* (Å)	4.4091(4)	6.3800(1)
*c* (Å)	8.8827(1)	13.4400(3)
*β* (°)	123.1346(7)	—
*x*_Ag1_	−0.0115(2)	0.7500
*y*_Ag1_	0.1737(1)	−0.0696(8)
*z*_Ag1_	0.3701(1)	0.2500
*x*_Ag2_	0.3095(8)	1.0000
*y*_Ag2_	0.8200(1)	0.2421(1)
*z*_Ag2_	0.9889(9)	−0.1231(3)
*x*_Te_	0.2758(8)	1.0000
*y*_Te_	0.1815(1)	0.2551(1)
*z*_Te_	0.2563(9)	−0.8854(2)

**Table 2:** Rietveld refinement results for 25.5 GPa.

Pressure (GPa)	25.5	
Space group	*Cmca*(No. 64)	*Pnma*(No. 62)
*a* (Å)	5.6399(9)	12.6999(9)
*b* (Å)	6.0000(0)	4.1479(9)
*c* (Å)	12.5799(9)	4.0000(0)
*x*_Ag1_	0.7500	0.2413(5)
*y*_Ag1_	0.056(4)	0.2500
*z*_Ag1_	−0.2500	0.4484(8)
*x*_Ag2_	1.0000	0.4115(3)
*y*_Ag2_	0.247(2)	0.7500
*z*_Ag2_	−0.0943(1)	0.4534(1)
*x*_Te_	1.0000	0.6006(2)
*y*_Te_	0.250(2)	0.7500
*z*_Te_	−0.9032(1)	0.0827(1)
