# Supplementary material for: Electronic Topological Transition in Ag2Te at High-pressure
Source: Sci Rep. 2015 Sep 30;5:14681. doi: 10.1038/srep14681 (PMC4588579; doi:10.1038/srep14681)
Supplement: Supplementary Information [file srep14681-s1.doc]

**Electronic Topological Transition in Ag_2_Te at High-pressure**

Yuhang Zhang, Yan Li^*^, Yanmei Ma, Yuwei Li, Guanghui Li, Xuecheng Shao, Hui Wang, Tian Cui, Xin Wang^*^, and Pinwen Zhu^*^

State Key Laboratory of Superhard Materials, College of Physics, Jilin University, Changchun 130012, China

^*^**Corresponding author:**

**E-mail:** zhupw@jlu.edu.cn.

xin_wang@jlu.edu.cn.

liyan2012@jlu.edu.cn.

**Supplementary Information**

**Fig. S1**: Rietveld refinement of (a) the *P*2_1_/*c* phase at 0.8 GPa, (b) the *P*2_1_/*c* phase at 2.0 GPa.

**
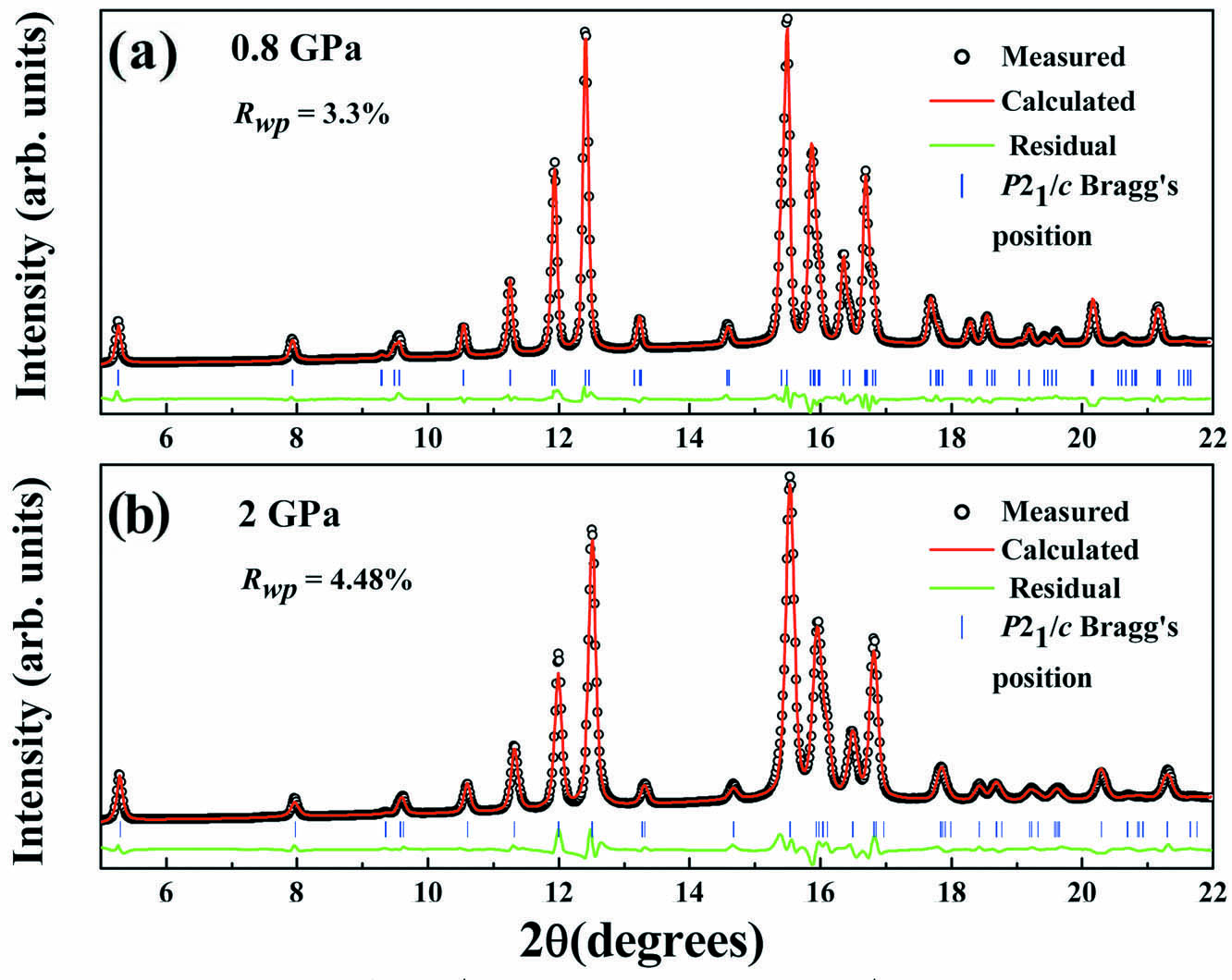
**

**Fig. S2**: Angle dispersive X-ray powder diffraction patterns of *Cmca* phase.

**
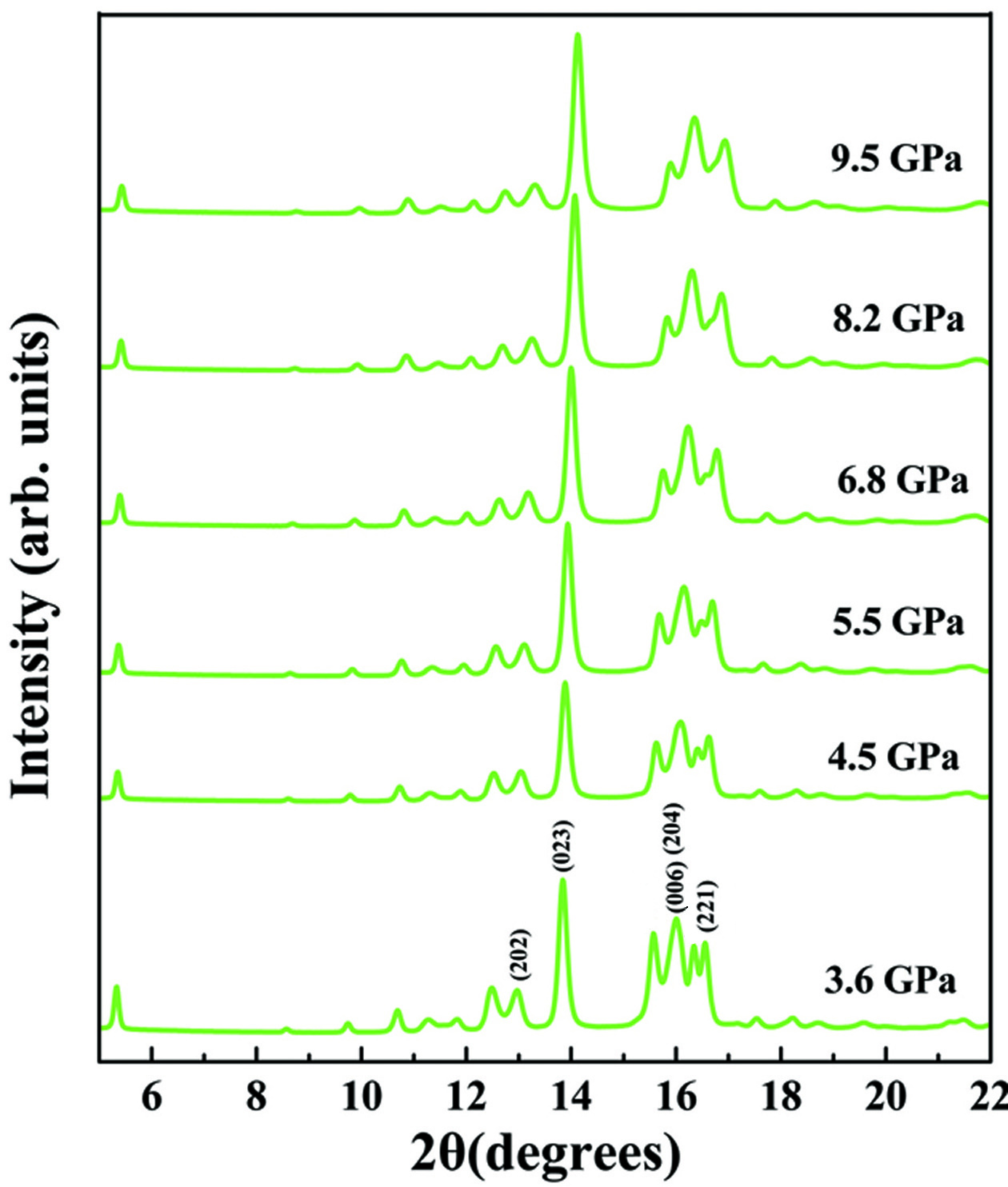
**

**Fig. S3**: Rietveld refinement result of 9.5 GPa.


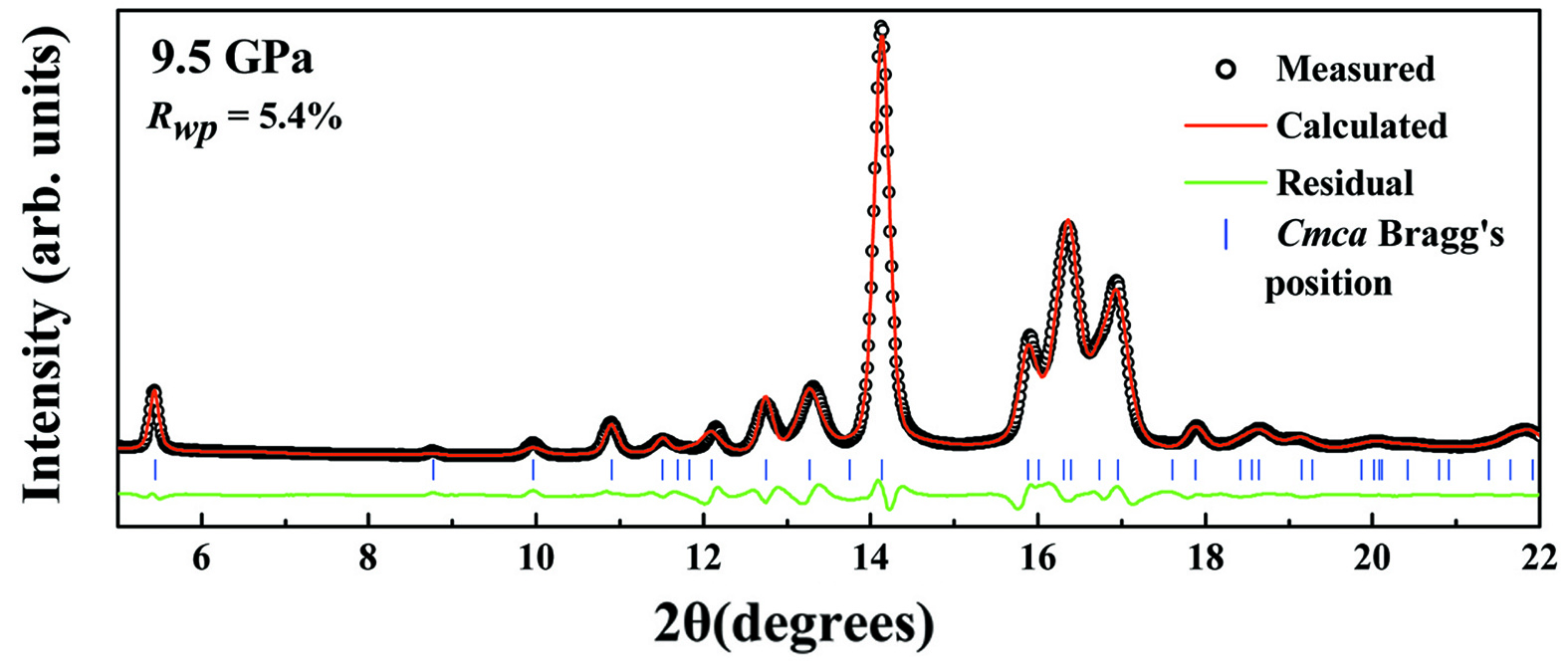


**Table S1.** Rietveld refinement results for 9.5 GPa.

| Pressure (GPa) | 9.5 |
| --- | --- |
| Space group | *Cmca* (No. 64) |
| *a* (Å) | 5.8838(6) |
| *b* (Å) | 6.1782(2) |
| *c* (Å) | 13.0470(0) |
| *β*(°) | - |
| *x*_Ag1_ | 0.7500 |
| *y*_Ag1_ | -0.0834(3) |
| *z*_Ag1_ | 0.2500 |
| *x*_Ag2_ | 1.0000 |
| *y*_Ag2_ | 0.1518(9) |
| *z*_Ag2_ | -0.09790(0) |
| *x*_Te_ | 1.0000 |
| *y*_Te_ | 0.3181(1) |
| *z*_Te_ | -0.8959(9) |

**Fig. S4**: Rietveld refinement results of (a) 2.2 and (b) 2.6 GPa, respectively. Inset shows the (023) peak of the *Cmca* phase.


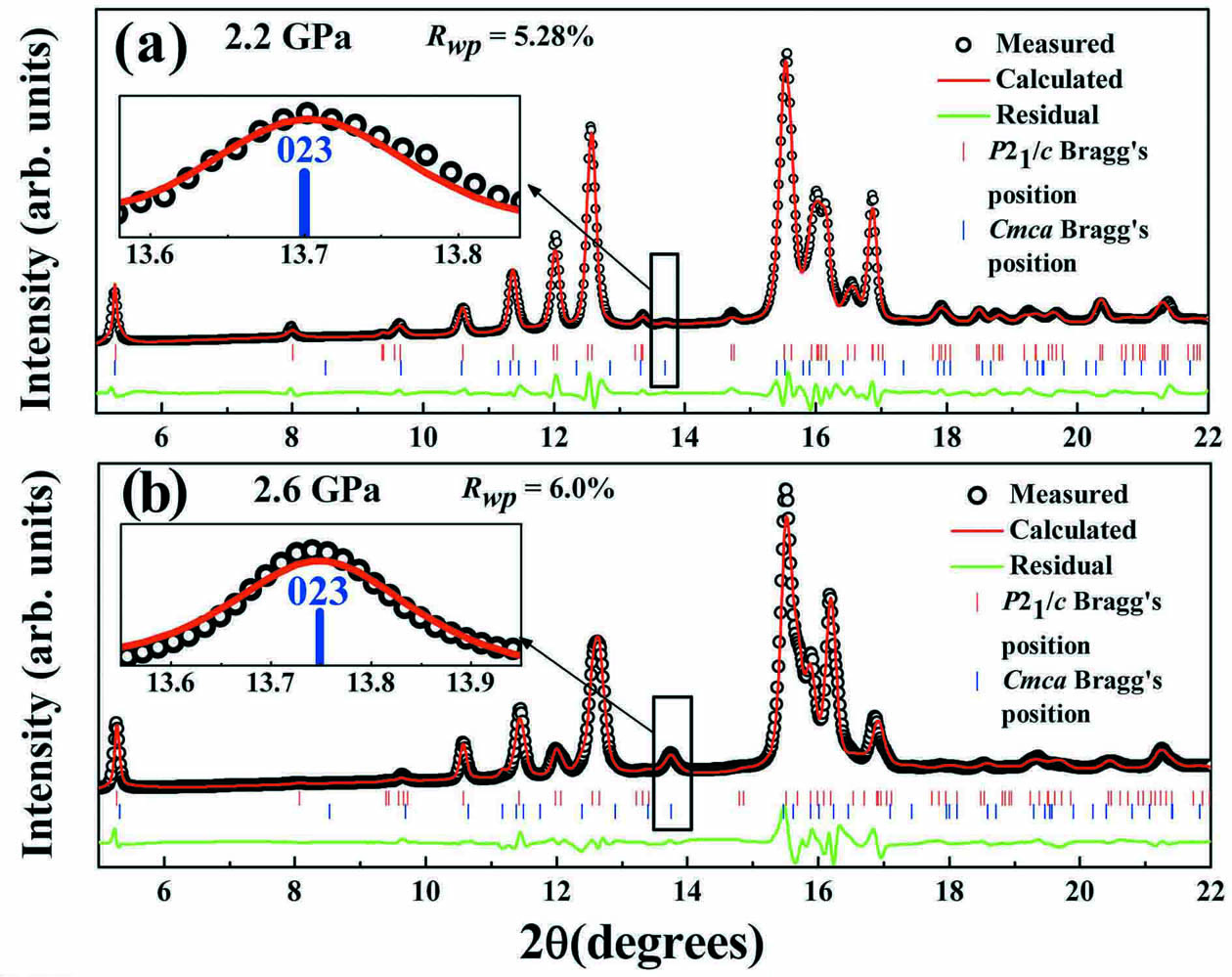


**Fig. S5**: (a) ADXRD patterns from 11.3 GPa to 22.5 GPa, and asterisk represents the peak of the *Pnma* phase. (b) ADXRD patterns from 11.3 GPa to 19.2 GPa, which are fitted by Origin software, and asterisk represents new peak.


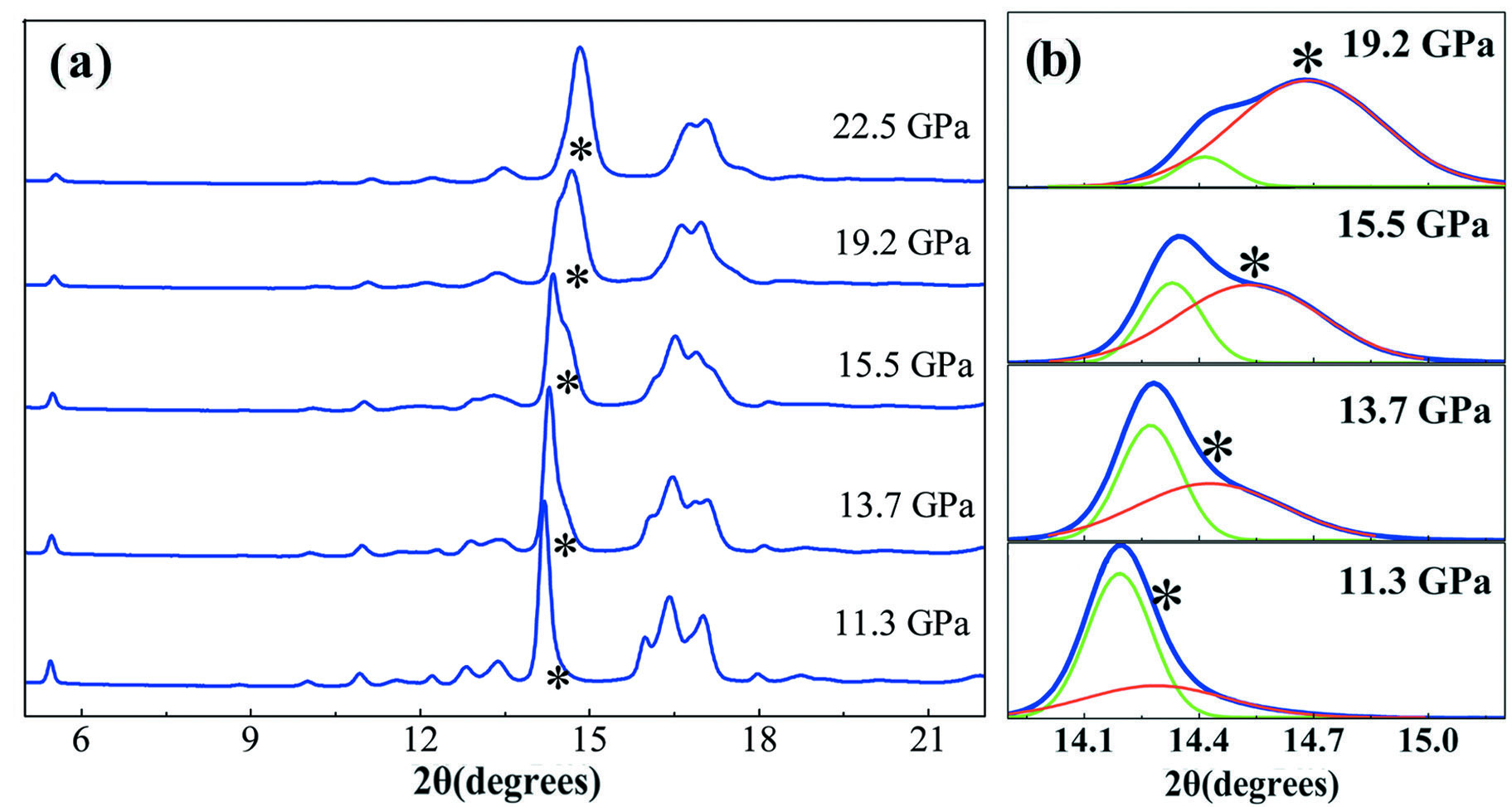


**Fig. S6**: Rietveld refinement result of 25.5 GPa.


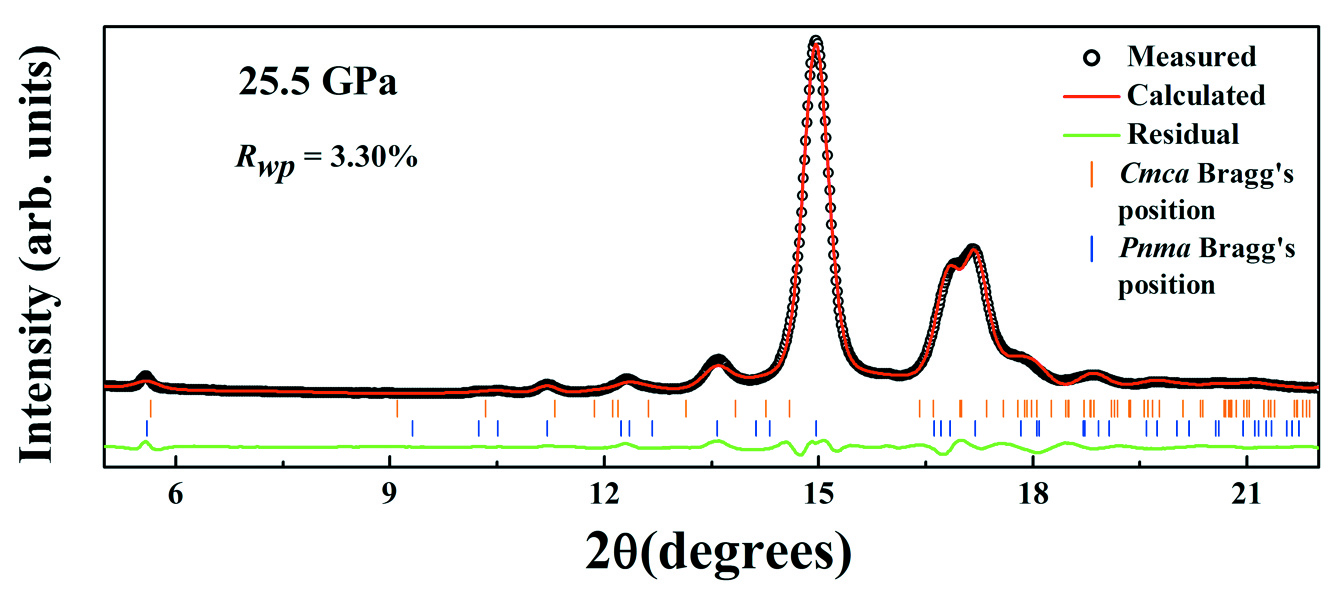


**Fig. S7**: Schematic representation of the *P*2_1_/*c*, *Cmca* and *Pnma* phase.


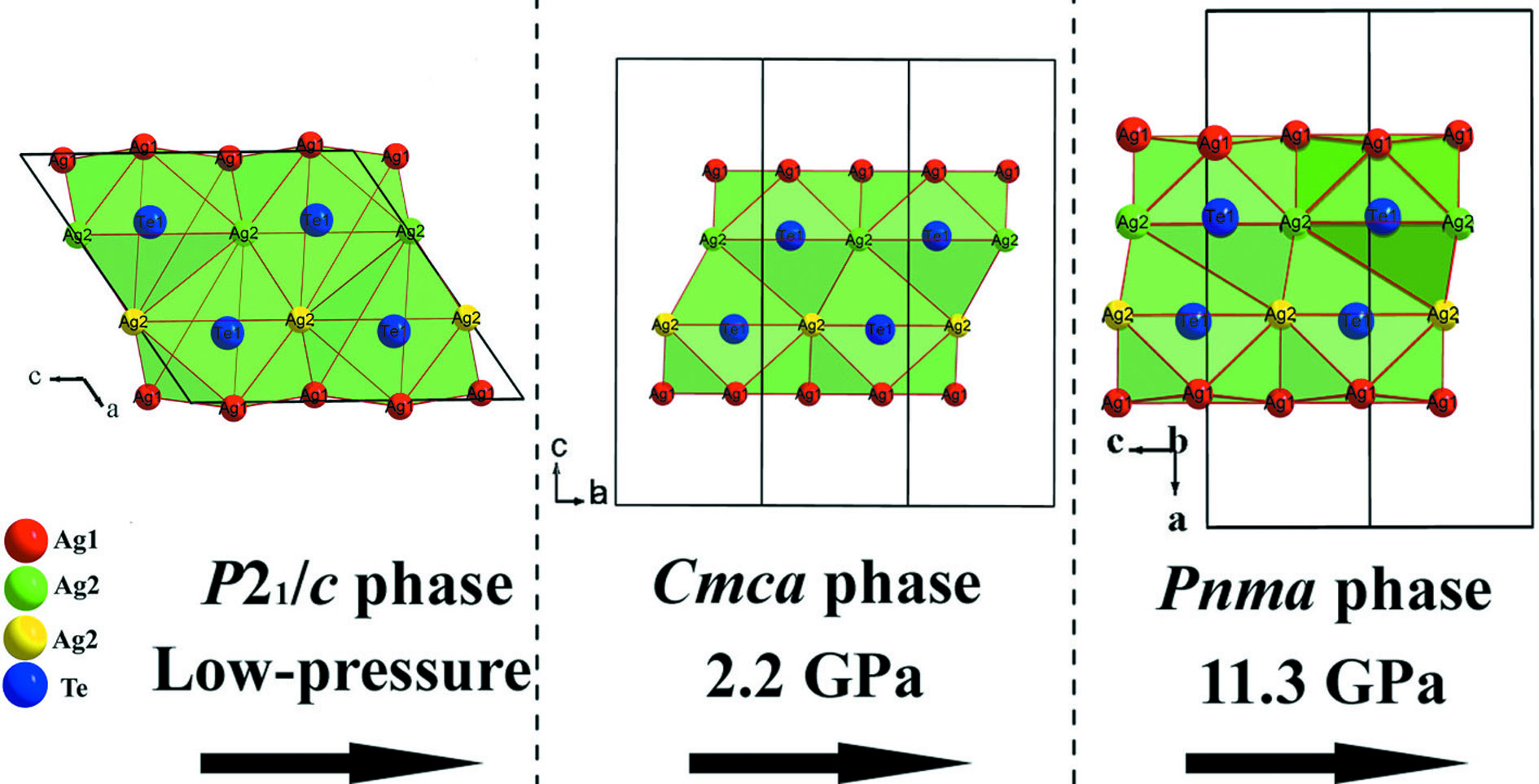


**Table S2.** Ag-Te bond lengths (Å) in *P*2_1_/*c* phase, *Cmca* phase and *Pnma* phase.

| *P*2_1_/*c*  (0.8 GPa) | *P*2_1_/*c*  (1.5 GPa) | *P*2_1_/*c*  (2.0 GPa) | *Cmca*  (4.5 GPa) | *Cmca*  (9.5 GPa) | *Pnma*  (25.5 GPa) |
| --- | --- | --- | --- | --- | --- |
| 2.855(4) | 2.824(4) | 2.849(7) | 2.816(6) | 2.815(8) | 2.922(7) |
| 3.013(8) | 2.834(5) | 3.002(9) | 2.860(6) | 2.815(8) | 2.740(8) |
| 2.885(2) | 2.860(4) | 2.889(6) | 2.860(6) | 2.835(7) | 2.740(8) |
| 2.946(0) | 2.931(6) | 2.931(8) | 2.947(7) | 2.900(8) | 2.747(1) |
| 2.959(5) | 2.945(9) | 2.976(4) | 2.947(7) | 2.900(8) | 2.987(2) |
| 2.966(2) | 2.984(9) | 2.924(8) | 2.954(8) | 2.910(8) | 2.987(2) |
| 2.964(7) | 2.987(9) | 2.947(9) | 3.011(6) | 2.950(8) | 2.787(2) |
| 2.845(0) | 3.013(8) | 2.848(0) | 3.011(6) | 2.950(8) | 2.787(2) |
|  |  |  | 3.342(8) | 3.270(8) | 2.821(9) |

**Fig. S8**: (a) Structure model, (b) Ag1 atoms arrangements, (c) coordination polyhedron shear glide, and (d) Ag2 atoms arrangements of the *P*2_1_/*c*, *Cmca* and *Pnma* phase.


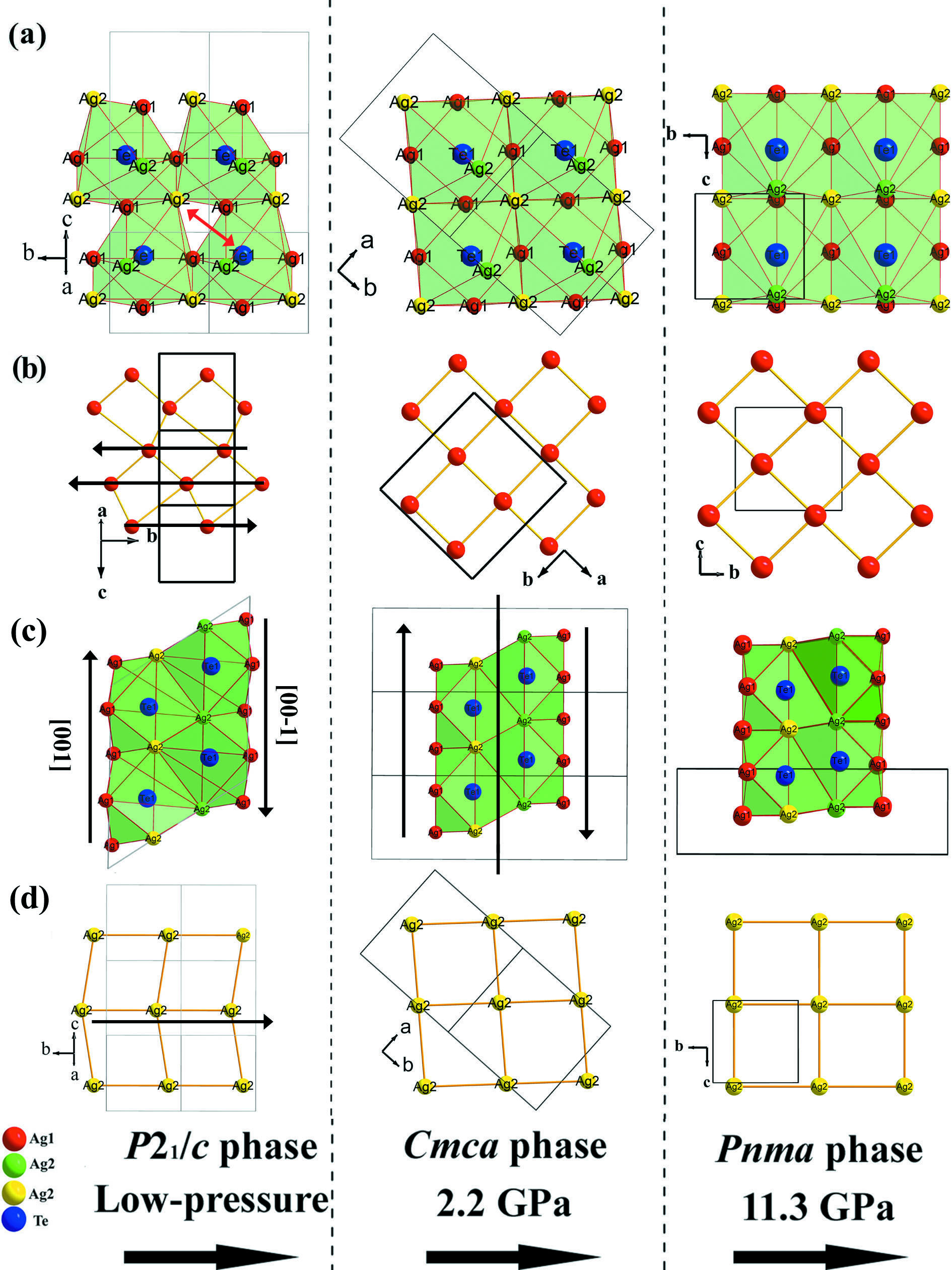


**Fig. S9**: (a) Pressure dependence of *K* in *P*2_1_/*c* phase. Lattice parameters of (b) *Cmca* and (c) *Pnma* phase with increasing pressure, respectively. Errors given by the GSAS EXPGUI package are smaller than the marker sizes.


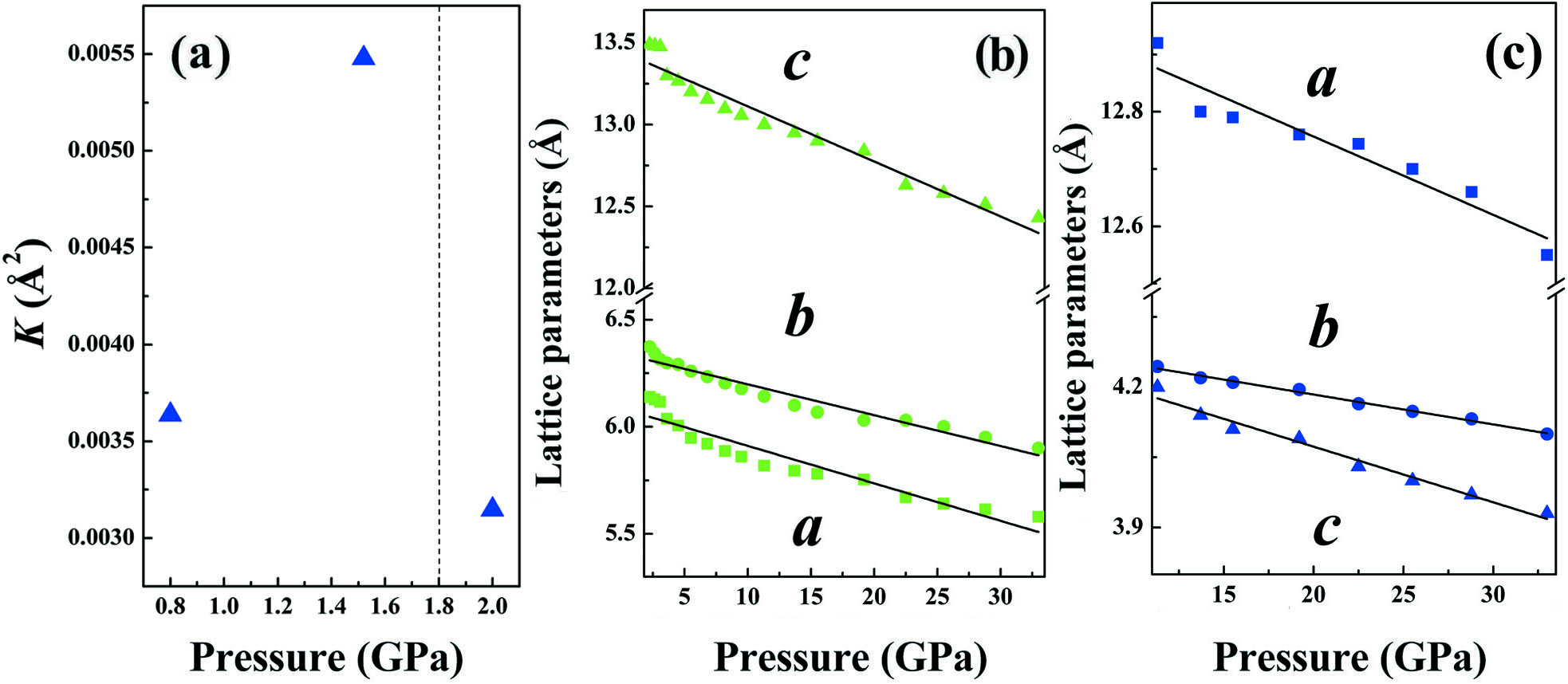


**Fig. S10**: Pressure dependence of per four formula units volume for the three phases of Ag_2_Te. Errors given by the GSAS EXPGUI package are smaller than the marker sizes for *P*2_1_/*c* and *Pnma* phase.

**
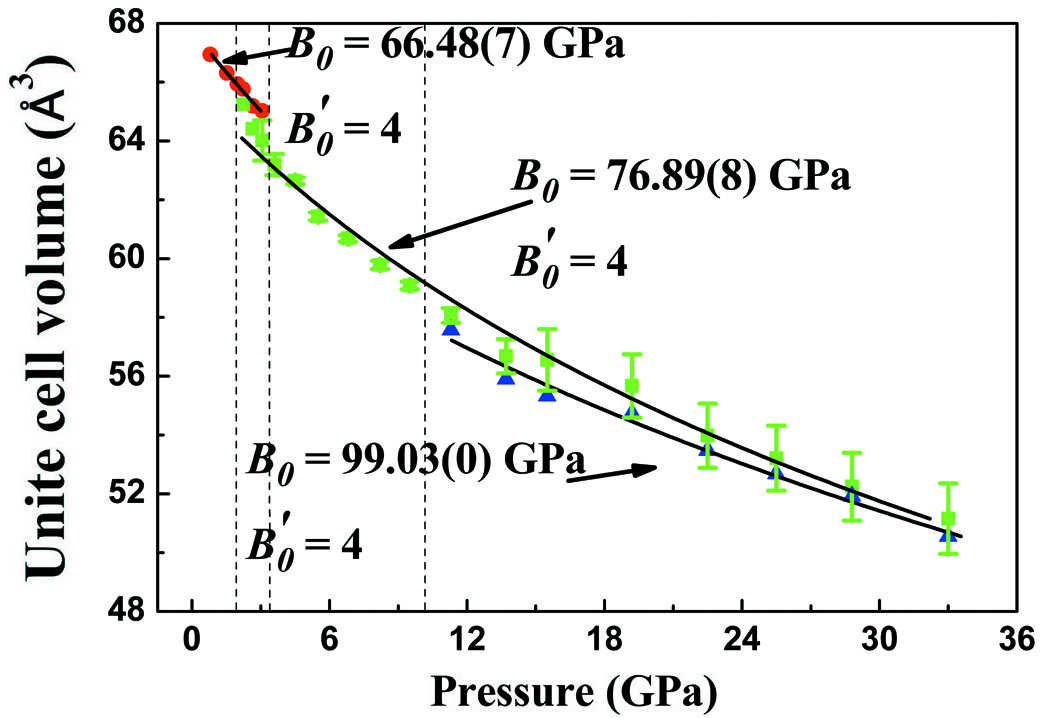
**

**Fig. S11**: Diffraction rings pattern of 7.8 GPa.

**
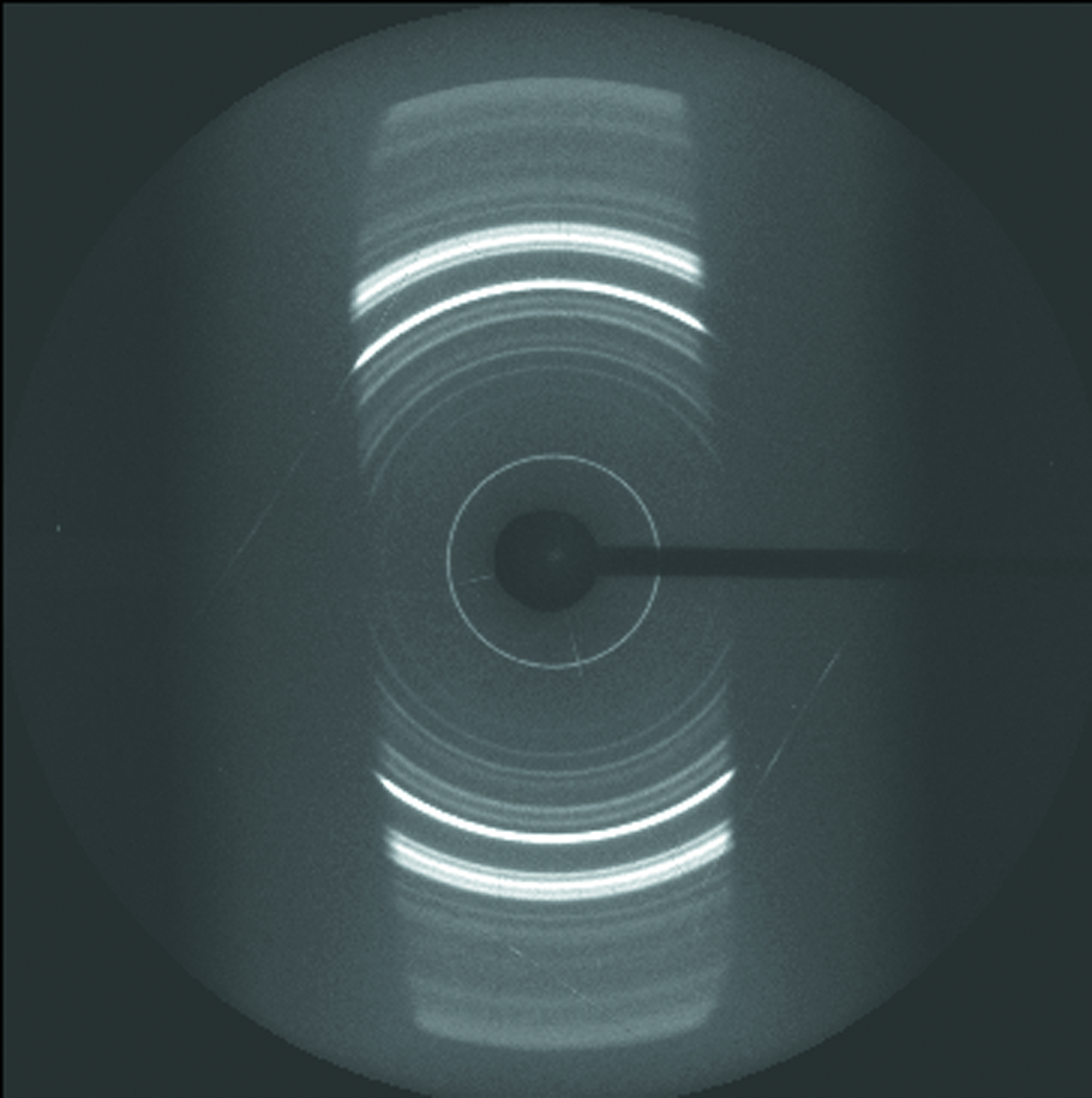
**

**Fig. S12**: EDX result of Ag_2_Te sample.

**
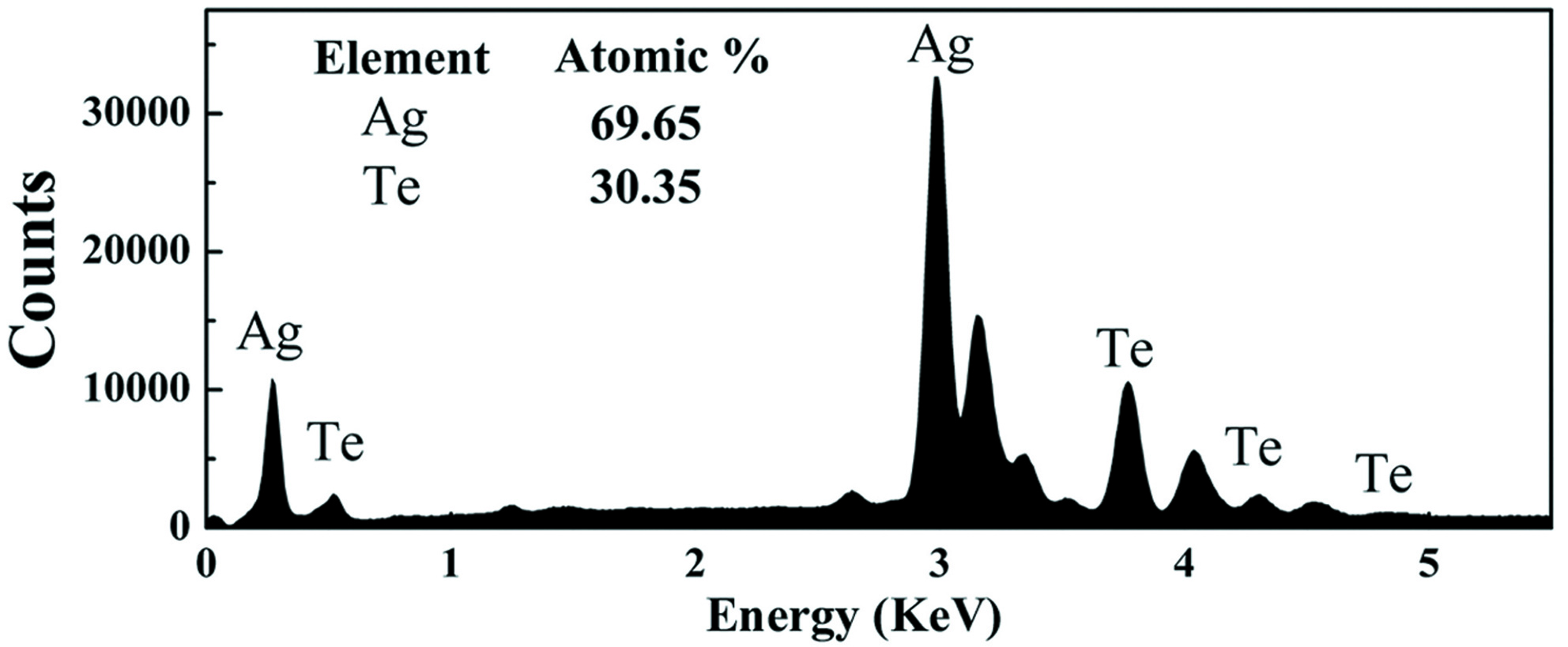
**
